# Image Adversarial Example Generation Method Based on Adaptive Parameter Adjustable Differential Evolution

**DOI:** 10.3390/e25030487

**Published:** 2023-03-10

**Authors:** Zhiyi Lin, Changgen Peng, Weijie Tan, Xing He

**Affiliations:** 1 State Key Laboratory of Public Big Data, College of Computer Science and Technology, Guizhou University, Guiyang 550025, China; 2 Key Laboratory of Advanced Manufacturing Technology, Ministry of Education, Guizhou University, Guiyang 550025, China

**Keywords:** adversarial example generation, adaptive differential evolution, neural network, image recognition

## Abstract

Adversarial example generation techniques for neural network models have exploded in recent years. In the adversarial attack scheme for image recognition models, it is challenging to achieve a high attack success rate with very few pixel modifications. To address this issue, this paper proposes an adversarial example generation method based on adaptive parameter adjustable differential evolution. The method realizes the dynamic adjustment of the algorithm performance by adjusting the control parameters and operation strategies of the adaptive differential evolution algorithm, while searching for the optimal perturbation. Finally, the method generates adversarial examples with a high success rate, modifying just a very few pixels. The attack effectiveness of the method is confirmed in CIFAR10 and MNIST datasets. The experimental results show that our method has a greater attack success rate than the One Pixel Attack based on the conventional differential evolution. In addition, it requires significantly less perturbation to be successful compared to global or local perturbation attacks, and is more resistant to perception and detection.

## 1. Introduction

Deep learning has achieved great success in many fields, particularly in computer vision, where neural network-based image recognition techniques are widely used in practical applications due to their high accuracy [1]. However, its application security issues have also attracted more and more attention, and adversarial example attack is one of the main security threats. According to the attacker’s grasp of the target model information, adversarial example attacks are classified as white-box attacks and black-box attacks. White-box attacks require the attacker to master the structure and parameters of the model, while black-box attacks do not need to know the model’s internal information. In the following, the development process of adversarial example methods will be described in detail according to the classification.

**White-box attacks.** In 2013, Szegedy et al. [2] first introduced the concept of adversarial examples. They demonstrated that adding tiny perturbations to the image can cause models to be classified incorrectly. The adversarial examples have prompted academics to question the trustworthiness of deep learning, as well as opened up research on adversarial attacks and defense. Goodfellow et al. [3] proposed the Fast Gradient Sign Method (FGSM) for computing perturbations on the hypothesis that deep learning models have linear properties in high-dimensional space. Subsequently, many enhancement schemes have been put up to address the FGSM’s flaws, including weak attack robustness and a lack of precision in the perturbation computation. For instance, Kurakin et al. [4] proposed the Basic Iterative Method, which optimizes the strength of perturbations by multiple small-step gradient updates. In addition, Dong et al. [5] proposed the Momentum Iterative Fast Gradient Sign Method (MI-FGSM) that integrates momentum into the iterative process to generate more migratory adversarial examples. After that, different from the above gradient-based solution methods, Moosavi-Dezfooli et al. [6] proposed the DeepFool method based on the concept of the hyperplane. It is used to determine the shortest distance between the decision boundary of the original sample and the adversarial example. To reduce the number of adversarial perturbations, Papernot et al. [7] proposed the Jacobian-based Saliency Map Attack to perturb the features that have the most impact on the classification results, to generate adversarial examples. Similarly, Phan et al. [8] used the Class Activation Map to find the image’s important features. By adding adversarial perturbations to these features, a well-migrated adversarial attack was achieved. In the face of the powerful defensive distillation at that time, Carlini and Wagner [9] proposed a C&W attack in L0, L2, and L∞ norms, and the model was completely ineffective against this defense. In addition, Zhang et al. [10] proposed a feature-based universal perturbation generation method by analyzing the feature differences between the original and adversarial examples. The method utilizes random source data instead of datasets, which further enhances the applicability of the algorithm. All of the aforementioned white-box attacks require the attacker to know the structure and parameters of the model. However, it is not practical for obtaining all the internal information of the target model in reality.

**Black-box attacks.** In 2017, Papernot et al. [11] trained alternative models to generate migratable adversarial examples, enabling the first black-box attack on adversarial examples without internal knowledge of the original model. Afterward, a method based on the migratory nature of adversarial examples has been proposed continuously [12,13,14]. Wu et al. [15] trained adversarial transformation networks to construct the most destructive perturbations and improved the transferability of adversarial examples. Zhou et al. [16] proposed Data-free Substitute Training (DaST) to obtain an alternative model without any real data. Based on DaST, Wang et al. [17] further proposed a diverse data generation module by stealing the knowledge of the target model to better generate the data distribution. To avoid the additional training and computational overhead caused by the alternative model, Narodytska et al. [18] proposed a Local Search Attack with local adversarial perturbation, which implemented only the local perturbation added to the image. In the more extreme limited scenario, Su et al. [19] proposed the One Pixel Attack (OPA) to further reduce the number of adversarial perturbations required for black-box attacks. 

The gradient estimation-based method is also one of the main methods of black-box attacks, and it can be combined with the alternative model to improve the efficiency of the attack [20]. Lin et al. [21] proposed the Black-box MI-FGSM, which approximated the gradient information of pixel points using the differential evolution technique. Wang et al. [22] used data distribution to identify important regions of black-box attacks and effectively approximated the model gradient information. Furthermore, many methods also take advantage of the strong noise immunity of boundary attacks to improve the efficiency of black-box attacks [23]. Shi et al. [24] proposed the Custom Adversarial Boundary Attack, which models the sensitivity of each pixel using the current noise and optimizes the adversarial noise for each image. Chen et al. [25] designed the Hop Skip Jump Attack to generate adversarial examples at the decision boundary, significantly improving the performance of the boundary attack.

In addition, Moosavi-Dezfooli et al. [26] proposed a universal adversarial perturbation computation method and demonstrated its excellent generalization performance on different datasets and network models. Later, to carry out targeted attacks on high-performance image classifiers, Sarkar et al. [27] proposed two black-box attacks based on the idea of generic perturbation: UPSET for creating generic perturbations for target classes and ANGRI for generating specific perturbations for different images. Gaussian distribution is frequently employed as a search distribution in black-box attacks, but it lacks flexibility. To address this issue, Feng et al. [28] transformed Gaussian distribution variables to another space that improves the capability and flexibility of capturing the inherent distribution of perturbations on benign samples.

This paper is devoted to the study of black-box attacks that add only very few perturbations to the images. Although the OPA requires only a few perturbations, it is based on the conventional Differential Evolution (DE) [29] algorithm. In the process of finding the optimal perturbation, the DE’s control parameters are subjective and fixed, and there is no operational strategy, which results in a low attack success rate. To address these issues, the primary contributions of this paper are as follows:

1. An image adversarial example generation method based on the DE is proposed in the black-box environment, which can achieve a higher attack success rate with only very few perturbations on the image.
2. An adaptive parameter adjustable differential evolution algorithm is proposed to find the optimal perturbation, which realizes the adaptive adjustment of the DE’s control parameters and operation strategies, and satisfies the dynamic requirements at different stages, so the optimal perturbation is obtained with a higher probability.
3. The experiments are conducted to confirm the efficacy of the proposed method. The results demonstrated that, compared to the OPA, our method can efficiently generate more adversarial examples. In particular, when expanded to three-pixel and five-pixel attacks, it significantly raises the attack success rate. In addition, the perturbation rate required by the proposed method is substantially lower than that of global or local perturbation attacks. The capacity to resist detection and perception in physical environments is further improved.

## 2. Related Work

The adaptability of adversarial attacks in physical environments has gradually increased over the past few years: from white-box attacks which require internal knowledge of the model, to black-box attacks which do not require knowledge of any network parameters, and from global image perturbation to local perturbation, even to one-pixel perturbation under extreme conditions.

The DE algorithm is a population-based global search technique, which is widely used for solving various complex optimization problems. For limited scenarios, Su et al. [19] first proposed the OPA based on DE. This method encodes the position information and intensity of the perturbed pixels and uses the DE to make the model feedback information guide the evolutionary direction of the adversarial perturbation. The optimal solution is obtained when the maximum number of iterations is reached or once there is convergence to a stable state. In contrast to previous adversarial attacks that aim to minimize the number of perturbations across the entire input image, the OPA focuses on controlling the number of perturbed pixels without limiting the intensity of their modification. However, the OPA is based on the conventional DE algorithm, which only implements a straightforward situation with a fixed mutation factor of 0.5 and no crossover operation, resulting in an attack success rate that needs to be improved. Following that, Su et al. [30] evaluated the effectiveness of using DE for producing the adversarial perturbation under different parameter settings. Under strict constraints that simultaneously control the number of pixels changed and the overall perturbation intensity, the experimental results showed that when the mutation factor and crossover probability were both 0.1, it could more effectively balance the success rate with the perturbation. However, this method still uses fixed parameter settings and does not take into account the dynamic requirements of the algorithm for the solution process.

Therefore, the fixed control parameters and operation strategies are not well adapted to the optimization issues in different scenarios, and changing selections of them based on the researcher’s subjective experience can easily have a great impact on the algorithm. So, different DE variations have been put forth, including the random control parameter setting [31] and adaptive setting [32,33]. It was found that the adaptive control parameter settings can significantly lower the risk of algorithm stagnation as well as can better adapt to optimization problems in complex situations [34]. Kushida et al. [35] proposed that Jing Adaptive Differential Evolution (JADE) would improve the efficiency of searching for the optimal adversarial perturbations. Wang et al. [36] used the particle swarm algorithm for the OPA’s optimization. The experimental results showed that the method can improve the success rate of the attack while maintaining the advantage of having a low degree of perturbation. In proposing a model-independent dual-quality assessment for adversarial machine learning, Vargas et al. [37,38] developed the Covariance Matrix Adaptation Evolution Strategy for a novel black-box attack, verifying the effectiveness of the adaptive strategy in improving the OPA performance. After that, Su et al. in [39] further showed the promises of evolutionary computation. It is both a way to investigate the robustness of DNNs as well as a way to improve their robustness through hybrid systems and the evolution of architectures.

The OPA and its optimization method both implement adversarial example attacks that modify only a very few pixels. However, they are based on conventional DE, which uses fixed control parameters and crossover strategies when finding the optimal perturbation, resulting in a low success rate. The proposed optimization methods are proposed later to verify the effectiveness of the adaptive strategy in improving the OPA performance. Therefore, an adversarial example generation method based on adaptive DE is proposed, which can effectively solve the deficiencies of OPA.

## 3. Problem Description

Assuming the original image I is an n-dimensional input vector $x=\left(x_{1},x_{2},\ldots ,x_{n}\right)$, where the scalar $x_{i}$ represents the pixel value, the probability that the classifier f correctly classifies x as class t is $f_{t}(x)$. The vector $p(x)=\left(p_{1},p_{2},\ldots ,p_{n}\right)$ is defined as the superimposed adversarial perturbation of the input vector x, which can alter the label of vector x from the original class t to the target class adv. In the case of the targeted attack, the target class adv is designated while for the non-targeted attack, it can be an arbitrary class as long as adv≠t. The element $p_{i}$ in the vector p(x) represents the perturbation added to the corresponding dimensional element $x_{i}$ of the input vector x. Specifically, $p_{i}=\left(x_{i},y_{i},r_{i},g_{i},b_{i}\right)$ contains the position and color information of the perturbed pixels. After that, the vector p(x) will be optimized by the proposed adaptive differential evolutionary algorithm to obtain the optimal adversarial perturbation of the original image. Assuming the optimal perturbation ${p(x)}^{*}$, the following conditions should be satisfied:

(1) $$\underset{{p(x)}^{*}}{\max\;}f_{adv}(x+p(x))$$

(2) $$subject\;to{\left\|p(x)\right\|}_{0}\leq L$$

where L in the restriction (subject to) is the maximum modification of the perturbation, and ${\left\|p(x)\right\|}_{0}$ denotes the modification of the vector p(x) under the L0-norm. Except for the elements $p_{i}$ that need to be modified, others in vector p(x) are left at zero. The combined Equations (1) and (2) show that the optimization objective of our method is to maximize the probability that the classifier f classifies the input vector x as the attack target adv. Ultimately, the optimal perturbation ${p(x)}^{*}$ is obtained, while the solution process restricts the maximum perturbation of the vector p(x) to not exceed the constraint L.

The majority of current global or local perturbation attacks do not strictly limit the number of perturbation pixels L, and fail to achieve the extreme situation of a very few pixels attack. The OPA uses the conventional DE to solve the optimal perturbation ${p(x)}^{*}$, and only a very few pixels are modified to attack successfully. However, the success rate is not high. Therefore, an optimized method can be proposed to search for the optimal perturbation ${p(x)}^{*}$. Generating adversarial examples with higher success rates while maintaining the advantage of low perturbation is the goal.

## 4. Proposed Method

**Figure 1:**
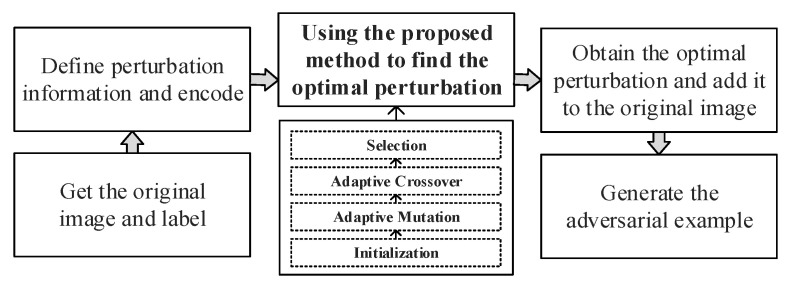
The flow chart of image adversarial example generation.

This section proposes an image adversarial example generation method based on adaptive parameter adjustable differential evolution to solve the OPA’s low success rate. In the process of utilizing the method to find the optimal perturbation, the control parameters and operation strategies in the DE are adaptively adjusted according to the number of iterations. Ultimately, by realizing the dynamic requirements of the solution process for the DE algorithm, the method effectively raises the success rate of the adversarial example attack and completes the OPA optimization. Figure 1 depicts the process flow for generating image adversarial examples based on the adaptive parameter adjustable DE, and the details are provided below.

### 4.1. Initialization

We encode the perturbation of the image $x=\left(x_{1},x_{2},\ldots ,x_{n}\right)$ as a candidate solution. Each candidate solution $p(x)=\left(p_{1},p_{2},\ldots ,p_{n}\right)$ contains a fixed number of perturbations $p_{i}$, one perturbation $p_{i}$ corresponds to modifying one pixel $x_{i}$. For an m-pixel attack, there is m perturbation information in p(x). Then, it will obtain the optimal perturbation ${p(x)}^{*}$ from the candidate solutions using the adaptive parameter adjustable DE. Note that for a clearer description of the method process, I(x) denotes the initialized perturbation vector, M(x) denotes the mutation perturbation vector, and C(x) denotes the crossover perturbation vector in the following. They are all variants of the perturbation p(x) in different stages of the evolution.

In the OPA [19], the initialized population (candidate solution) is randomly generated in the solution space. To prevent the aggregation problem of data samples owing to simple random sampling, this paper applied Latin hypercube sampling to generate the initial candidate solutions, which made the individual sample (perturbation) more uniform and comprehensive. To do this, the candidate solution size is set to NP and the perturbation’s dimension to D. The Latin hypercube sampling method is used to obtain the parameter information ${I(x)}_{i,j,0}$, which constitutes the initialized perturbation vector ${I(x)}_{i,0}$:

(3) $${I(x)}_{i,j,0}=LHS({min}_{j},{max}_{j})$$

where {i=1,2,...,NP}, j={1,2,...,D}. LHS is the Latin hypercube sampling method. When performing sampling, any dimension j of the perturbation vector ${I(x)}_{i,0}$ should be restricted to its range of values $\left[{min}_{j},{max}_{j}\right)$. Meanwhile, the initial means of the mutation factor and crossover probability are set to 1 and 0.9 for $\mu_{F}$ and $\mu_{CR},$ respectively. During each generation of evolution, the sets $S_{F}$ and $S_{CR}$ need to be created to store the mutation factor F and crossover probability CR of the successful adversarial perturbation vector in the current generation.

### 4.2. Adaptive Mutation

Mutation operation is beneficial for enhancing the diversity of the population. The mutation operation can produce more diverse perturbations in the candidate solutions for adversarial example generation. However, in the mutation operation of conventional DE, the fixed mutation factor and mutation strategy can limit the performance of the algorithm in the evolutionary process. Thus, we proposed an adaptive mutation operation to deal with this problem. At the early stages of the solution process, the effectiveness of perturbation I(x) is poor, and the range of the candidate solution space is expanded by a larger mutation factor F. While the DE/rand/1 mutation strategy is used to randomly select perturbations to reduce the probability I(x) of being trapped in the local optimum, at the later stages, the effectiveness of I(x) is enhanced, the convergence speed of the algorithm is improved by a smaller F, and the DE/best/1 mutation strategy is used to guide I(x) to evolve toward the optimal solution.

In adaptively adjusting the mutation factor, the initial F follows a normal distribution with a mean $\mu_{F}$ and standard deviation 0.05:

(4) $$F_{i}=randn_{i}\left(\mu_{F},0.05\right)$$

From Equation (4), the distribution of F is impacted by modifying $\mu_{F}$. The $\mu_{F}$ is adaptively adjusted according to the number of iterations, which in turn affects the value of F. Finally, the F is made to satisfy the dynamic demand of the perturbation vector at different evolutionary stages. The rules for calculating the mean $\mu_{F}$ are as follows:

(5) $$\mu_{F}=\left(1-c_{1}\right)\times \mu_{F}+c_{1}\times \frac{\sum_{F\in S_{F}}\;F^{2}}{\sum_{F\in S_{F}}\;F}-c_{2}\times \pi^{\frac{g}{G}}$$

where $c_{1}$ and $c_{2}$ are constants, G is the maximum number of iterations, and g is the current number of iterations. The second term in Equation (5) is the Lehmer mean function, which is helpful for propagating larger values of F as a way to improve the progress rate [32]. Meanwhile, the set $S_{F}$, which stores the mutation factors of previously successful adversarial examples, is used to guide the generation of new $\mu_{F}$.

In adaptively adjusting the mutation strategy, we provide a new method to realize the dynamic selection of DE/rand/1 and DE/best/1 at various stages of evolution. First, five perturbations from the current generation are chosen at random. Three of them generate ${I(x)\prime }_{i,g}$ according to DE/rand/1, and the remaining two generate ${I(x)\prime \prime }_{i,g}$ according to DE/best/1:

(6) $$\left\{\begin{array}{l} {I(x)\prime }_{i,g}={I(x)}_{r1,g}+F_{i}\times \left({I(x)}_{r2,g}-{I(x)}_{r3,g}\right)\\ {I(x)\prime \prime }_{i,g}={I(x)}_{best,g}+F_{i}\times \left({I(x)}_{r4,g}-{I(x)}_{r5,g}\right)\; \end{array}\right.$$

where r1,r2,r3,r4,r5 are unequal integers chosen at random from the set {1,2,...,NP}, ${I(x)}_{best,g}$ is the optimal perturbation in the current generation g, and is selected randomly in the 0th generation. Thereafter, the ${I(x)}_{best,g}$ will be updated according to the effectiveness of the new perturbation vectors. According to the number of iterations, ${I(x)\prime }_{i,g}$ and ${I(x)\prime \prime }_{i,g}$ jointly generate the mutation perturbation ${M(x)}_{i,g}$ of the current generation g:

(7) $${M(x)}_{i,g}={I(x)\prime }_{i,g}\times \left(1-\frac{g}{G}\right)+{I(x)\prime \prime }_{i,g}\times \frac{g}{G}$$

### 4.3. Adaptive Crossover

Crossover operation can improve the individual variability and population diversity. For adversarial example generation, the crossover probability CR primarily affects the degree of information exchange between the initialized perturbation ${I(x)}_{i,g}$ and the mutation perturbation ${M(x)}_{i,g}$. For the absence of the crossover operation in the OPA, we proposed an adaptive crossover operation to optimize the solution process. In the early stages of the solution process, a large CR is used to ensure that more components of the perturbation from mutation perturbations M(x) improve the speed of the solution. Then, a relatively small CR is used in the later stages to maintain the accuracy of the final optimization result.

Therefore, similar to how the adaptive mutation factor is created, the initialized CR follows a normal distribution with a mean $\mu_{CR}$ and standard deviation 0.05:

(8) $${CR}_{i}=randn_{i}\left(\mu_{CR},0.05\right)$$

The solution process is then measured by the number of iterations, causing the $\mu_{CR}$ to gradually decrease, which in turn affects the distribution of the CR and achieves its adaptive adjustment. Similarly, the set $S_{CR}$, which stores the crossover probabilities of previously successful adversarial examples, is used to guide the generation of the new $\mu_{CR}$. The rule for calculating the $\mu_{CR}$ is as follows:

(9) $$\mu_{CR}=\left(1-c_{3}\right)\times \mu_{CR}+c_{3}\times \frac{\sum_{CR\in S_{CR}}\;CR}{size\left(S_{CR}\right)}-c_{4}\times \pi^{\frac{g}{G}}$$

where $c_{3}$ and $c_{4}$ are constants. Then, the initial perturbation ${I(x)}_{i,g}$ needs to perform the crossover operation with the mutation perturbation ${M(x)}_{i,g}$ to increase the diversity of the candidate solutions. Each dimension of the crossover perturbation ${C(x)}_{i,j,g}$ is obtained as follows:

(10) $${C(x)}_{i,j,g}=\left\{\begin{array}{l} {M(x)}_{i,j,g},\;\;if\;rand[0,1]\;<\;CR_{i}\;\;or\;j\;=\;randint(1,D)\\ {I(x)}_{i,j,g},\;\;\;\;\;\;\;\;\;\;\;\;\;\;\;\;\;otherwise \end{array}\right.$$

where j=randint(1,D) ensures that at least one component originates from ${M(x)}_{i,g}$, preventing the scenario where all ${I(x)}_{i,g}$ are transmitted to ${C(x)}_{i,g}$ and no new perturbations can be efficiently generated.

The aforementioned adaptive operations can ensure that each generation of control parameters and operation strategies change dynamically with the evolutionary process during the iterative solution. This enables the algorithm to have the corresponding global search capability and local optimization capability at different stages, while taking into account the convergence speed and solution accuracy. Ultimately, this improves the success rate of adversarial attacks.

### 4.4. Selection

Before the selection operation, it is necessary to calculate the crossover perturbation and the initial perturbation’s effectiveness. The more effective the perturbation is at minimizing the predefined adversarial example loss function $f_{\mathrm{loss}\;}$, the smaller the loss value. Thus, the selection operation is as follows:

(11) $${I(x)}_{i,g+1}=\left\{\begin{array}{l} {C(x)}_{i,g},\;\;if{\;f}_{\mathrm{loss}\;}({C(x)}_{i,g})<f_{\mathrm{loss}\;}({I(x)}_{i,g})\\ {I(x)}_{i,g},\;\;\;\;\;\;\;\;\;\;\;\;otherwise \end{array}\right.$$

The more effective perturbation can participate in the next iteration. The F and CR of the perturbation are stored in the set $S_{F}$ and $S_{CR}$ respectively, which guide the update of the $\mu_{F}$ and $\mu_{CR}$ of the next generation. Meanwhile, the current optimal perturbation ${I(x)}_{best,g}$ is compared and updated based on its effectiveness. The above operations are repeated until all perturbation in the current candidate solution space is traversed.

After that, the optimal perturbation of the current generation is added to the original image, and it is determined whether it satisfies the attack success condition:

(12) $$f_{adv}\left(x+{I(x)}_{best,g}\right)>f_{t}(x)$$

**Figure 2:**
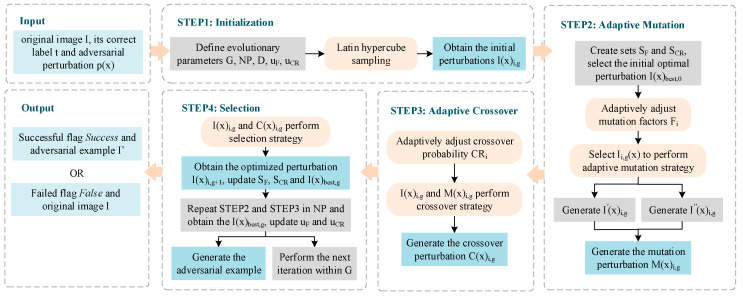
The process of finding adversarial perturbations using the adaptive parameter adjustable differential evolution method.

If the condition is met, ${I(x)}_{best,g}$ is the optimal perturbation ${p(x)}^{*}$, and ${p(x)}^{*}$ is added to the original image to successfully generate the adversarial example. If not, all perturbations found in the current candidate solution space with better effectiveness will be used as the initial candidate perturbations for the next iteration. Then, the iteration will continue until the adversarial example generation condition is satisfied, or the predetermined maximum number of iterations is reached. Figure 2 illustrates the process of finding adversarial perturbations by using adaptive parameter adjustable differential evolution (APADE). Algorithm 1 shows the method applied to adversarial example generation.
**Algorithm 1:** Image Adversarial Example Generation Method Based on Adaptive Parameter Adjustable Differential Evolution(1) **Input:** Original image I and its correct label t(2) **Output:** Adversarial example I’, or original image I.(3) $x=\left(x_{1},x_{2},\ldots ,x_{n}\right)\leftarrow I$(4) $p(x)=\left(p_{1},p_{2},\ldots ,p_{n}\right)$(5) flag, image ← **APADE**(x, t, p(x))(6) **if** flag == Success(7)   **return** I′ (8) **else**(9)   **return** I(10)**end if**

In the theoretical analysis, the time complexity of our method is O(G×NP×D), i.e., it depends on the maximum number of iterations G, the range of candidate solutions NP, and the perturbation dimension D. Since the crossover operation is not used in the OPA, its time complexity is O(G×NP), which is less than that of our method. Additionally, the OPA only uses the fixed mutation operation, and the adaptive mutation and crossover operations in our method increase the running time of the algorithm. These computational costs are unavoidable for achieving higher attack success rates than the OPA. Therefore, further optimization of our method to improve the efficiency of the solution can be left as future work.

## 5. Experiment and Analysis

In this section, we aim to validate the proposed adversarial example attack method based on adaptive differential evolution, as well as analyze the experimental results. Ultimately, it is compared with other adversarial example generation methods.

### 5.1. Experimental Setup

Three typical neural networks were trained for the datasets CIFAR10 [40] and MNIST [41] in this paper: ResNet [42], Network in Network (NinN) [43], and VGG16 [44]. The CIFAR10 consists of 60,000 32 × 32 color images split into 10 classes, including 50,000 training images and 10,000 test images. In addition, the MNIST consists of 70,000 28 × 28 grayscale images divided into 10 classes, with 60,000 training images and 10,000 test images. In the training process, the number of training rounds was set between 100 and 200 depending on the convergence degree of the model, and 128 training samples were taken in each round. Table 1 shows the final classification accuracy of the models on the three datasets. 

**Figure 3:**
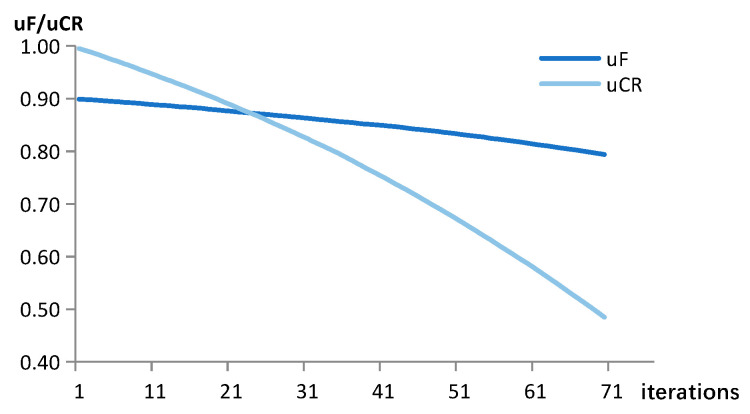
The trend of $\mu_{F}$ and $\mu_{CR}$ with the number of population iterations.

During the attack phase, images from the CIFAR10 or MNIST test datasets were randomly selected for each of the attacks on the three neural networks. After confirming that these images had been correctly identified in the corresponding network, they were utilized to carry out both the targeted and non-targeted attacks. In the experiments, the adaptive differential evolutionary algorithm was used to generate adversarial examples. The algorithm performed one iteration of the candidate solution size NP=400 and the maximum number of iterations of the evolutionary solution G=100. In the setting of the perturbation dimension D, D=5 in the CIFAR10 and D=3 in the MNIST. For the adaptive mutation and crossover operations, the initial means $\mu_{F}=1$ and $\mu_{CR}=0.9$. The trends of $\mu_{F}$ and $\mu_{CR}$ when performing a successful attack are shown in Figure 3 (the curved graph is smoothed to some extent).

**Figure 4:**
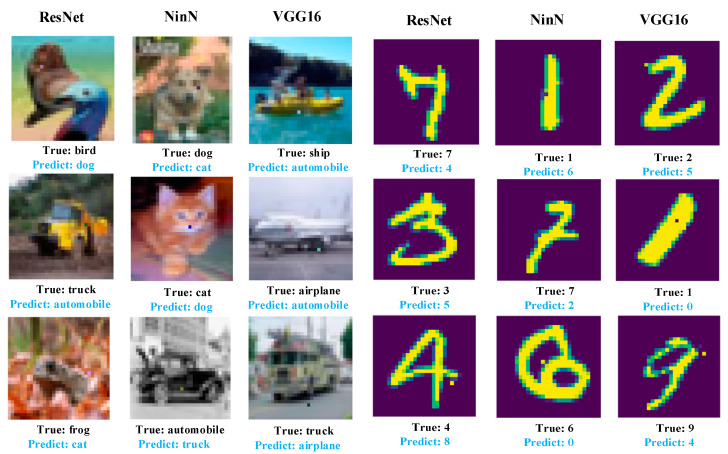
The adversarial examples generated by our proposed method. By perturbing a very few number of the images’ pixels, our method successfully fooled three neural networks—ResNet, NinN, and VGG16. (**a**) Adversarial examples generated for the CIFAR10. (**b**) Adversarial examples generated for the MNIST.

After completing the above main setup, we began the adversarial example attack experiment. Meanwhile, we extended the experiment to 3 and 5 pixels to compare with the OPA, and confirmed the impact of the adversarial perturbation number on the attack success rate. Figure 4 displays the visualized results of the experiment.

### 5.2. Analyze the Success Rate of Attack

There are two different ways to define the attack success rate. In the targeted attack, it is the probability that the current image is perturbed to the rest of the specified target class that is not itself. In the non-targeted attack, simply perturb the current class to the rest of the non-self class. The three neural networks are attacked in two different ways, and each attack way perturbs 1 pixel, 3 pixels, and 5 pixels of the image. Table 2 displays the final attack success rates.

According to Table 2, ResNet, NinN, and VGG16 were susceptible to the adversarial examples produced by perturbing very few pixels. In terms of the attack type, the ResNet trained on the CIFAR10 achieved an 84% success rate for a 5-pixel non-targeted attack, which is nearly 32% higher than the corresponding targeted attack. This shows that the non-targeted attacks had higher success rates than targeted attacks. As the number of perturbed pixels increased, so did the success rates and the targeted class for both assault methods, proving that the number of modified pixels had a positive correlation with the experiment’s success rate.

Additionally, in conjunction with the data in Table 1, we monitored the impact of the network classification accuracy on the attack success rates. The attack success rate will normally be higher when the accuracy is low, and it will be reduced as the accuracy increases. Of the three experimental networks, ResNet had the best classification accuracy for the CIFAR10. As a result, it had a rather low attack success rate, and compared to the other two networks, it was more robust to this attack. However, when the accuracy was not much different, the attack results were subject to unstable fluctuations.

### 5.3. Analyze the Sensitivity of Attack

**Figure 5:**
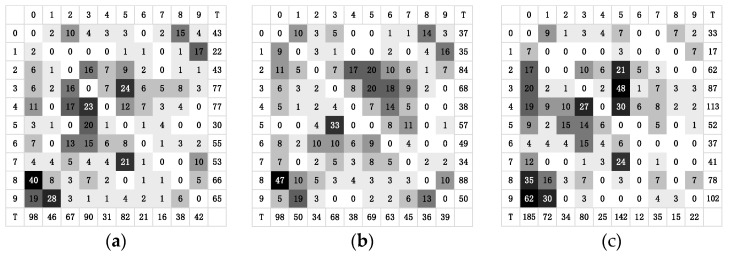
Heat maps of the number of times each class in the CIFAR10 was perturbed to other classes when attacking the networks. (**a**) Heat map of the attack on ResNet. (**b**) Heat map of the attack on NinN. (**c**) Heat map of the attack on VGG16.

**Figure 6:**
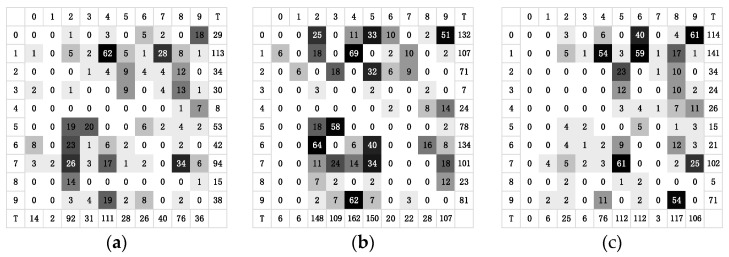
Heat maps of the number of times each class in the MNIST was perturbed to other classes when attacking the networks. (**a**) Heat map of the attack on ResNet. (**b**) Heat map of the attack on NinN. (**c**) Heat map of the attack on VGG16.

The sensitivity of attack is defined as the ease with which the original class of the image can be perturbed to other classes. In the experiment, we recorded the number of times each original class was perturbed to various other classes. The total value was used as the quantitative data of the class attack sensitivity after calculating the total number of perturbations in the class. Thus, the more times a class was perturbed to other classes, the more sensitive it was to this attack, where, in the incorrect attack in the targeted attack, the perturbed class of the original image was recorded as its actual perturbed situation. Figure 5 and Figure 6 illustrate the number and corresponding total value of times each class in the CIRFAR10 and MNIST was perturbed to other classes when the ResNet, NinN, and VGG16 are attacked, respectively. In Figure 5, the number from 0 to 9 in the first row and the first column represent, respectively, the classes: airplane, automobile, bird, cat, deer, dog, frog, horse, ship, and truck. The capital T indicates the total number of times.

Figure 5 and Figure 6 indicate that some classes were more vulnerable than others. For instance, the cat in CIFAR10 was relatively simple to perturb to other classes. The ship in NinN even was perturbed to all other target classes, while the automobile was relatively more difficult to disturb. Number 1 of the MNIST was more vulnerable than number 8. In practice, malicious users are more likely to take advantage of the more sensitive classes, leaving the entire model vulnerable to attack. In fact, for the less sensitive classes, their data points are difficult or even impossible to perturb to other classes. Studying the essential reasons for these data points’ resistance to modification could lead to innovative adversarial defense strategies.

Analyzing the individual classes, the ship in CIFAR10 could readily become an airplane but barely ever the frog, and the number 1 in MNIST could easily be perturbed as number 4 but hardly perturbed as number 3. Su et al. stated in [19] that the OPA can be viewed as a data point perturbation along an axis’ parallel direction in n dimensions. Similar to this, a 3-pixel or 5-pixel attack will cause the data points in the corresponding dimension’s cube to move. Thus, a few-pixel attack is essentially a perturbation of a low-dimensional slice in the input space. The experimental results demonstrated that moving the data points’ vertical directions in the n-dimensional space could create adversarial examples of various classes. In essence, these adversarial examples shared data points belonging to the same original class. The ease with which the original class could be perturbed to a certain target class was dependent on the size of the decision distance between the original class and the target class.

### 5.4. Comparison of Experimental Results

In the following, the experimental results will be compared with the current typical adversarial example attack methods from the two aspects of: the success rate of the attack and the disturbance amount. The advantages of our method are illustrated through the comparison of the data.

**Figure 7:**
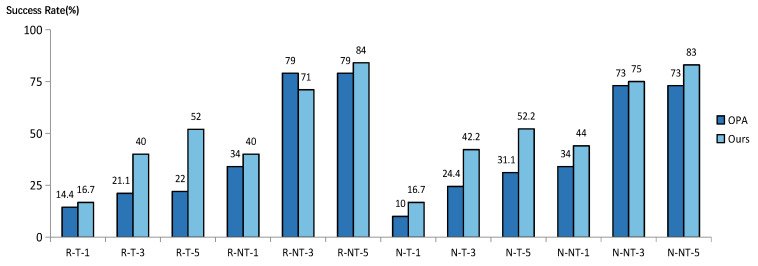
The success rate of our method compared with OPA on CIFAR10-based ResNet and NinN.

Although the OPA implements a few pixel attacks, it is based on the conventional DE algorithm. Because there is no crossover method and only fixed control parameters are employed when searching for the optimal perturbation, the attack success rate still needs to be improved. Therefore, we proposed an adversarial example generation method based on the adaptive DE, which not only achieved very few pixel attacks, but also effectively overcame the deficiencies of OPA. For comparison, we selected the study’s rather complete experimental data. The experiments were conducted with the same network, dataset, and amount of perturbation. Finally, Figure 7 shows the comparison of our method with the OPA in terms of the attack success rate.

Where R stands for ResNet, N for NinN, T for targeted assault, NT for the non-targeted attack, and the digits 1, 3, and 5 for 1-pixel, 3-pixel, and 5-pixel attack, respectively, on the horizontal axis, Figure 7 demonstrates that our method generally had a higher success rate than the OPA and that this improvement is significant. In particular, the success rate was increased by 30% with the targeted 5-pixel attack on ResNet (R-T-5). Additionally, our method had a better improvement effect on the targeted attack success rate from the attack strategies, with an average increase in about 16%. The aforementioned results demonstrated that our method of finding the optimal perturbation based on adaptive DE can effectively satisfy the dynamic requirements of the global search capability and local optimization capability of the algorithm in different solving stages. So, it can obtain the optimal solution with a higher probability, and achieve a better success rate on adversarial example attacks.

Our method, as one of the optimization methods of the OPA, was also compared with other optimization schemes such as Jing Adaptive Differential Evolution (JADE) [35], Particle Swarm-based Optimization (PSO) [36], and Covariance Matrix Adaptation Evolution Strategy (CMA-ES) [37,38] (described in detail in Section 2). These methods also aimed to implement the adversarial example attack by modifying the image with only very few pixels. Therefore, we compared the success rate of the attack with them for the same dataset, network, and number of modified pixels. Table 3 shows the success rate of our method and other optimization schemes in attacking the ResNet and NinN for the CIFAR10.

As can be seen from Table 3, the attack success rate of our method outperformed other methods overall, which is attributed to the advantage of our method in solving optimal perturbations using the adaptive differential evolution algorithm.

In comparison with existing adversarial example attack methods, we selected some typical methods in terms of the amount of perturbation required for a successful attack, the environment, and the type of attack. These methods include the Fast Gradient Sign Method (FGSM) [3], DeepFool (DF) [6], Jacobian-based Saliency Map Attack (JSMA) [7], and Local Search Attack (LSA) [18] (described in detail in Section 1). Table 4 shows the comparison of these methods with ours for attacks on CIFAR10 and MNIST datasets, respectively.

Where the perturbation rate is defined as the percentage of the number of modified pixels to the total number of pixels, Table 3 shows that compared with the existing typical methods, our method significantly reduces the amount of perturbation required in the attack. It even needs only 0.1% perturbation to attack successfully and is more resistant to perception and detection. From the analysis of the environment and the principle of adversarial example generation, our method mainly has the following advantages:

1. Our method does not use gradient information for the optimization and does not require the objective function to be differentiable or previously mastered. Therefore, it belongs to the black-box attack and is more practical than gradient-based methods, in reality.
2. Compared with gradient descent or greedy search algorithms, our method is relatively less affected by local optima and can find the global optimal perturbation with a higher probability.

Our method is a further study of very few pixel attacks. The performance described above demonstrated that the current adversarial example attack technology has a higher attack success rate and concealment, and the security threat to the deep model is increasingly serious. Therefore, by showing the analysis of the principles of adversarial example generation methods in the extreme environment, we hope that it can provide new ideas for the research of corresponding adversarial example defense and detection techniques. Furthermore, the robustness of the model against adversarial example attacks is enhanced.

## 6. Conclusions

This paper proposes an image adversarial example generation method based on adaptive parameter adjustable differential evolution. In the process of seeking the optimal perturbation, the control parameters and operation strategies in the algorithm are adaptively adjusted according to the number of iterations. It satisfies the dynamic demand for the global search capability and local optimization capability of the algorithm in different solving stages. The adversarial example attack with a high success rate is achieved with only very few pixel perturbations. The experimental results demonstrate that our method, with only 0.48% of perturbations, achieves a success rate of over 80% for a neural network trained on CIFAR10 and has a good attack effect when the dataset is moved to MNIST. Compared with the OPA based on the conventional differential evolution, our adaptive method can realize a higher attack success rate while maintaining limited conditions. Compared with previous global or local perturbation attacks, our method simply requires less perturbation at the time of attack success and has stronger resistance to perception and detection.

The following research directions for the adversarial example technology can be taken into consideration:

1. There are numerous variants of the DE, some of which enhance the variation strategy mechanism [45,46], and combine the DE with other intelligent algorithms [47,48]. If the appropriate DE variants are selected in the context of certain issues, it would be possible to achieve adversarial attacks that are more effective and precise.
2. Of course, adversarial defense will also be a key area of study in the future. The majority of the conventional defense strategies have either been successfully cracked or proven ineffective [49,50,51]. Adversarial example detection techniques, which are a supplementary defense strategy, fail to completely distinguish the original samples from adversarial examples [52].

In fact, adversarial attacks and defenses are a mutual game process. The generation of attack methods will promote the development of defense strategies, and later these defense strategies may be broken by new attack techniques. Therefore, the study of attack algorithms can lay the foundation for proposing more effective defense strategies. In particular, exploring the adversarial example technique with a high success rate and low perturbation can provide more insight into the model structure and algorithm’s working mechanism. Further, designing adversarial defense algorithms that are more effective and robust should be completed to make the model more secure and controllable.

## Figures and Tables

**Table 1:** Image classification accuracy of ResNet, NinN, and VGG16 models trained on CIFAR10 and MNIST datasets.

	ResNet	NinN	VGG16
**CIFAR10**	92.31%	86.07%	78.28%
**MNIST**	99.15%	99.01%	95.82%

**Table 2:** Success rates of attacks on three networks based on CIFAR10 and MNIST.

Dataset	Non-Targeted Attack			Targeted Attack			Network
	1-Pixel	3-Pixel	5-Pixel	1-Pixel	3-Pixel	5-Pixel	
CIFAR10	40%	71%	84%	16.67%	40%	52.22%	ResNet
	44%	75%	83%	16.77%	42.22%	52.22%	NinN
	52%	77%	84%	33.33%	61.11%	74.44%	VGG16
MNIST	4%	40%	60%	—	10%	27.78%	ResNet
	4%	26%	51%	10%	26.67%	34.44%	NinN
	10%	37%	56%	8.89%	23.33%	31.11%	VGG16

**Table 3:** For CIFAR10, our method compared with other OPA optimization schemes in terms of attack success rate.

Method	Number of Modified Pixels			Network
	1	3	5	
Ours	40%	71%	84%	ResNet
JADE	32.5%	77.5%	-	ResNet
PSO	31%	59%	65%	ResNet
CMA-ES	12%	52%	73%	ResNet
Ours	44%	75%	83%	NinN
CMA-ES	18%	62%	81%	NinN

**Table 4:** Comparison of different adversarial example generation methods in terms of perturbation (the number of pixels and the corresponding perturbation rate), environment, and attack type when attacking CIFAR10 and MNIST datasets.

Dataset	Method	Perturbation	White/Black-Box	Attack Type
CIFAR10	Ours	1 (0.10%)	Black-box	Adaptive DE-based
	Ours	3 (0.29%)	Black-box	Adaptive DE-based
	Ours	5 (0.49%)	Black-box	Adaptive DE-based
	LSA	38 (3.75%)	Black-box	Greedy search-based
	DF	307 (30%)	White-box	Gradient-based
	FGSM	1024 (100%)	White-box	Gradient-based
MNIST	Ours	1 (0.13%)	Black-box	Adaptive DE-based
	Ours	3 (0.38%)	Black-box	Adaptive DE-based
	Ours	5 (0.64%)	Black-box	Adaptive DE-based
	LSA	18 (2.24%)	Black-box	Greedy search-based
	JSMA	32 (4.06%)	White-box	Gradient-based
	FGSM	1024 (100%)	White-box	Gradient-based

## Data Availability

The data used to support the findings of this study will be available from the corresponding author upon request.
